# The associations of the national health and productivity management program with corporate profits in Japan

**DOI:** 10.4178/epih.e2022080

**Published:** 2022-09-23

**Authors:** Yuichiro Yano, Hiroshi Kanegae, Koichi Node, Atsushi Mizuno, Akira Nishiyama, Hiromi Rakugi, Hiroshi Itoh, Kaori Kitaoka, Naoki Kashihara, Fumiaki Ikeno, Ichiro Tsuji, Kunio Okada

**Affiliations:** 1 Department of Advanced Epidemiology, Noncommunicable Disease (NCD) Epidemiology Research Center, Shiga University of Medical Science, Otsu, Japan; 2 Department of Family Medicine and Community Health, Duke University, Durham, NC, USA; 3 Genki Plaza Medical Center for Health Care, Tokyo, Japan; 4 Department of Cardiovascular Medicine, Saga University, Saga, Japan; 5 Department of Cardiology, QI Center, St. Luke’s International Hospital, Tokyo, Japan; 6 Department of Pharmacology, Faculty of Medicine, Kagawa University, Kagawa, Japan; 7 Department of Geriatric and General Medicine, Osaka University Graduate School of Medicine, Suita, Japan; 8 Department of Endocrinology, Metabolism and Nephrology, Keio University School of Medicine, Tokyo, Japan; 9 Department of Nephrology and Hypertension, Kawasaki Medical School, Kurashiki, Japan; 10 Division of Cardiovascular Medicine, Department of Medicine, Stanford University School of Medicine, Stanford, CA, USA; 11 Division of Epidemiology, Department of Health Informatics and Public Health, Graduate School of Medicine, Tohoku University School of Public Health, Miyagi, Japan; 12 Non-Profit Organization Kenkokeiei, Tokyo, Japan

**Keywords:** Health and productivity management, Smoking, Lifestyle

## Abstract

**OBJECTIVES:**

Using a dataset from a survey on national health and productivity management, we identified health and productivity factors associated with organizational profitability.

**METHODS:**

The Ministry of Economy, Trade and Industry conducted an annual survey on Health and Productivity Management between 2014 and 2021. We assessed the associations of organizational health and productivity management using survey questions collected in 2017 and 2018, with the rate of change in profits from 2017 and 2018 to 2020. We identified factors associated with organizational profitability using eXtreme Gradient Boosting, and calculated SHapley Additive exPlanation (SHAP) values for each factor.

**RESULTS:**

Among 1,593 companies (n= 4,359,834 employees), the mean age of employees at baseline was 40.3 years and the proportion of women was 25.8%. A confusion matrix for evaluating model performance had an accuracy of 0.997, precision of 0.993, recall of 0.997, and area under the precision-recall curve of 0.999. The most important factors related to an increase in corporate profits were the percentage of current smokers (SHAP value, 0.121), per-employee cost of health services (0.084) and medical services (0.050); the percentage of full-time employees working in sales departments (0.074) and distribution or customer service departments (0.054); the percentage of employees who slept well (0.055); and the percentage of employees within a company who regularly exercised (0.043).

**CONCLUSIONS:**

Employees’ lifestyle-related health risk factors and organizations’ management systems were associated with organizational profitability. Lifestyle medicine professionals may demonstrate a significant return on investment by creating a healthier and more productive workforce.

## INTRODUCTION

Employers, organizations, and national economies are under increasing global pressure to improve performance, optimize efficiency, and deliver value [1]. The size of the labor force in Japan is declining due to an aging population and fewer children being born. If this trend continues, the country’s gross domestic product will inevitably decline. The Japanese government, businesses, and citizens are working hard to solve this problem. One of the solutions to avoid a decline in the Japanese economy is to increase human performance by optimizing how to leverage the human assets of an organization. Human performance is expected to be greater when people are physically and emotionally able to work and have the desire to work [2-4]. Higher levels of human performance lead to higher levels of productivity, which in turn potentially lead to higher profits [5].

In Japan, the Ministry of Economy, Trade and Industry (METI) has defined essential elements of an integrated health protection and health promotion model to create healthier, high-performing workforces. The program is known in Japan as *Kenko* (health) *Keiei* (business). As a part of the efforts to achieve one of the goals set in Japan’s Revitalization Strategy, in 2014 the METI and the Tokyo Stock Exchange (TSE) decided to jointly designate qualifying companies as Health and Productivity Stock Selections—companies enhancing value through health and productivity management. This program evaluates companies’ health and productivity management efforts using a scoring system based on an annual survey. Companies ranked in the top 20% of all respondents are certified as outstanding health and productivity management organizations. The Health and Productivity Stock Selection designation is expected to incentivize more companies to undertake health and productivity management practices to enhance employees’ vitality and productivity, leading to increased mid-term to long-term performance and corporate value as well as improving investors’ understanding and evaluation of organizations [6,7].

Through this program, METI and TSE encourage companies to further enhance efforts for health and productivity management. However, the associations between specific approaches in health and productivity management and organizational profitability have not yet been studied. Identifying strongly correlated approaches could help companies make better decisions regarding the development and implementation of employee health programs. These decisions would consequently improve performance and productivity, increase value for investors, and strengthen the national economy. Using a dataset that includes quantitative and qualitative responses from a survey on health and productivity management, we identified health and productivity factors associated with organizational profitability.

## MATERIALS AND METHODS

METI conducted an annual survey on Health and Productivity Management between August 2014 and October 2021. The survey included approximately 60 questions in 5 domains: (1) the prioritization of health and productivity goals in the organization’s management philosophy and policies; (2) processes for improving health and productivity; (3) specific systems and metrics for implementing health-conscious management approaches; (4) metrics for assessing and improving these approaches; and (5) compliance with laws, regulations, and risk management priorities [8,9]. The survey questions have been expanded every year, and thus there is variance in the available information between 2014 and 2021. A financial performance measure (i.e., profit) was included beginning in 2017. Therefore, we used the dataset collected from 2017 to 2020 for our analyses. The survey was conducted at of the end of each fiscal year (FY). The FY2017 survey covered the period from April 2016 to March 2017, the FY2018 survey covered the period from April 2017 to March 2018, and The FY2020 survey covered the period from April 2019 to March 2020. The coronavirus disease 2019 pandemic has hurt the economy and negatively impacted many companies’ financial performance. Therefore, we did not use data collected for FY2021 (i.e., data from April 2020 to March 2021).

When we defined baseline data collected for FY2017 as the exposure and the rate of change in profits from FY2017 to FY2020 as the outcome, the number of companies with corresponding data for use in the analyses was 842. When we defined baseline data collected for FY2018 as the exposure and the rate of change in profits from FY2018 to FY2020 as the outcome, the number of companies with corresponding data for use in the analyses was 751. When the same company responded in both 2017 and 2018, we used the response from 2017. We combined and analyzed both sets of data (i.e., n= 1,593), since the content in the survey was largely similar between 2017 and 2018. We did not use data from companies that did not complete the survey in both FY2017 and FY2018 and did not use surveys in which the content was not directly related to health and productivity management. Supplementary Material 1 summarizes the survey questions used for the current analysis.

### Statistical analysis

Companies’ financial performance may be affected by the number of regular/non-regular employees. For each company, we calculated its profit divided by the number of employees each year and evaluated the rate of change in profit per employee between baseline (2017 or 2018) and 2020. The distribution of increases in corporate profit is shown in Supplementary Material 2. We categorized all companies into quartiles based on the rate of change in profit after baseline, and defined the highest-quartile group as comprising companies that had an increase in corporate profit.

The high multicollinearity among variables in the dataset limits accuracy and reliability in identifying associated factors. Therefore, we used eXtreme Gradient Boosting (XGB) [10], a classification approach that is robust with regard to multicollinearity. The XGB approach enabled us to rank factors in order of their strength of association with corporate profitability.

**Figure 1.**
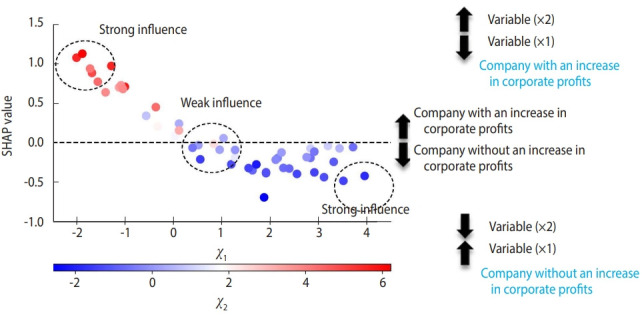
Feature importance related to an increase in corporate profits using the eXtreme Gradient Boosting algorithm. SHapley Additive exPlanations (SHAP) plot showing relative feature importance. A SHAP summary plots order features based on their importance. Each plot is made up of individual points from the dataset with a higher value being red and a lower value being blue. If the plots on side of the middle line are more red or blue, this indicates that the values are increasing or decreasing, respectively, moving the prediction in that direction.

To determine the hyperparameters of classifiers (specified by the analyst for optimizing the model performance), we used a 5-fold stratified cross-validation randomization and grid search approach. A hyperparameter is a parameter that is used to control the learning process (e.g., number of random forest trees) as opposed to parameters, the weights of which are learned during the training (e.g., variable weights). Tuning hyperparameters refers to iterations of the model architecture after seting the parameter weights to achieve the ideal performance. Five-fold cross-validation-based accuracy, defined as (true positive+true negative)/ (true positive+false negative+false positive+true negative) was evaluated for all possible combinations of hyperparameters. We selected the combination of hyperparameters that optimized the accuracy of model building. The hyperparameters considered for optimization included subsample, reg_lambda, reg_alpha, min_child_weight, max_depth, gamma, and colsample_bytree. Then, for each combination of these values, the model was fitted to 4 training folds and evaluated using a remained test fold. Finally, the average of the results was considered. We used SHapley Additive exPlanations (SHAP) values, which could explain black-box machine learning algorithms [11]. SHAP estimates values to determine each feature’s contribution to the output of the model (Figure 1). The SHAP value delineates between a condition being true (value > 0.0, companies with an increase in corporate profits) and it being false (value < 0.0, companies without an increase in corporate profits). The more a specific value of a sample influences the composition of the model, the farther the point will migrate away from zero on the y-axis. If the value of a sample does not influence the model, it will reside near or at 0 on the y-axis. In the example, a lower value of “X” and larger value of “Z” are highly predictive of the company having an increase in corporate profits, with these values strongly influencing the model “Y.” In the current study, we listed the top 10 features (factors) associated with corporate profitability. To evaluate the accuracy of the model, we provided a confusion matrix for binary classification, including measures of overall classification accuracy and the area under the precision-recall curve (AUC-PR). We defined a model with the AUC-PR curve > 0.90 as robust and acceptable. All analyses were performed with Python version 3.9.7 (Python Software Foundation, Wilmington, DE, USA).

### Ethics statement

We conducted this study under the oversight of the Ethical Committee of the Shiga University of Medical Science (RRB21-053-2) and in accordance with the principles of the Declaration of Helsinki. Because of the deidentified nature of records (i.e., survey questions for companies), informed consent was not obtained for each individual in accordance with the Ethical Guidelines for Medical and Health Research Involving Human Subjects in Japan.

## RESULTS

The database included survey results from 1,593 companies (n= 4,359,834 employees; Table 1). The types of industries represented among the 1,593 companies included construction (n= 69, 4.3%), food (n = 53, 3.3%), chemistry (n = 65, 4.1%), electrical manufacturing (n= 94, 5.9%), transportation equipment (n= 61, 3.8%), shipping (n=55, 3.5%), telecommunication (n=198, 12.4%), wholesale (n= 117, 7.3%), retail (n= 179, 11.2%), financial services (n= 139, 8.7%), professional services (n= 202, 12.7%), other (n = 361, 22.7%). Employees’ mean ± standard deviation age at baseline was 40.3±3.4 years, the proportion of women was 25.8%, and the mean length of service for employees was 14.2± 4.9 years.

### eXtreme Gradient Boosting model performance

Table 2 shows an evaluation of model performance. The confusion matrix for evaluating model performance showed an accuracy of 0.997, precision of 0.993, recall of 0.997, AUC of 0.999, and AUC-PR of 0.999.

### Feature importance assessment

**Figure 2.**
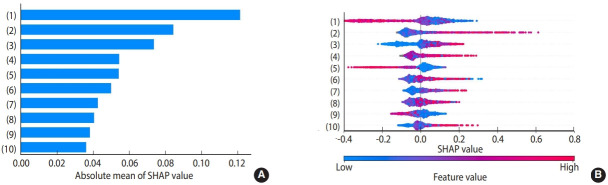
Absolute mean values (A) and feature importance (B) of the top 10 features with the strongest correlations to corporate profits. (A) The absolute mean values for the 10 features that most strongly correlated with corporate profits are shown. The features and the corresponding survey question noted in Supplementary Material 1 are: (1) Percentage of employees who were current smokers (Q22); (2) Per-employee cost of health care (Q59); (3) Percentage of full-time employees assigned to a sales department (Q7); (4) Percentage of employees who slept well (Q24); (5) Percentage of full-time employees assigned to a distribution or customer service department (Q7); (6) Per-employee cost of medical services (Q59); (7) Percentage of employees who exercised regularly (Q23); (8) Per-employee annual welfare expenses (Q60); (9) Number of employees who left the company during the fiscal year (Q5); and (10) Percentage of employees assigned to an “other role” department(s) (Q7). (B) SHapley Additive exPlanations (SHAP) summary plots for the above 10 features. Each plot is made up of individual points from the dataset with a higher value being red and a lower value being blue. If the plots on one side of the middle line are more red or blue, this indicates that the values are increasing or decreasing, respectively, moving the prediction in that direction. For example, a higher percentage of current smokers in a company, as shown in the top feature, i.e., (1), is associated with a lower probability of being a company with an increase in corporate profits.

Figure 2 illustrates the relative importance of features related to an increase in corporate profits. Based on SHAP values from the XGB model, the top 10 features with the strongest correlations to corporate profits included the percentage of current smokers (SHAP value, 0.121), per-employee cost of health services (0.084) and medical services (0.050), the percentage of full-time employees working in sales departments (0.074) and distribution or customer service departments (0.054), the percentage of employees who slept well (0.055), the percentage of employees within a company who regularly exercised (0.043), the amount of per-employee annual welfare expenses (0.041), the number of employees who left the company during the fiscal year (0.038), and the percentage of employees assigned to “other role” departments (0.036).

## DISCUSSION

Using data from a survey on national health and productivity management, we identified the top 10 factors in health and productivity management associated with an increase in organizational profitability, including employees’ lifestyle-related health risk factors (i.e., smoking, sleep, and exercise) and the organization’s management philosophy, policies, and systems. Prior associations of employees’ lifestyle-related health risk factors with worker productivity and healthcare costs have been reported [12-18]. However, these associations were assessed among a relatively small number of companies, and in those studies it is unclear whether employees’ lifestyle-related health risk factors (e.g., the percentage of current smokers) within a company were associated with organizational profitability. Furthermore, when assessing the associations of employees’ lifestyle-related health risk factors with organizational profitability, no studies have integrated information regarding the organization’s philosophy, policies, and specific systems and metrics related to health and productivity management. The current study extends existing knowledge by demonstrating that employees’ lifestyle-related health risk factors (i.e., smoking, sleep, and exercise) were more closely correlated with organizational profitability than the organization’s management philosophy, policies, and systems.

The mechanisms affecting employees’ lifestyle-related health risk factors and organizational profitability in the current study remain uncertain. However, there is a wealth of research connecting lifestyle-related health risk factors to worker productivity [19]. For example, worker productivity losses associated with smoking occur due to absenteeism, disability, and presenteeism associated with illness, smoking breaks, increased on-the-job accidents, and worker compensation costs, as well as the effect of secondhand smoke on coworkers [15,20]. Increasing physical activity has been shown to be linked to improvements in health conditions, a person’s overall physical and mental well-being, and reduced absenteeism [21-23]. Workplace sleep health promotion programs, including sleep hygiene, yoga, physical activity, and cognitive-behavioral therapy for insomnia, may increase employees’ sleep duration and subsequent daytime performance [24]. In a prior study of 4 Japanese companies (3,126 women and 12,350,9224 men), the monetary value due to absenteeism was US$520 per person per year, that of presenteeism was US$3,055, and medical/pharmaceutical expenses were US$1,165 [17]. Future studies of health and productivity management should include assessments of worker health, absenteeism, disability and presenteeism costs, which could address the mechanisms underlying the association of employees’ lifestyle-related health risk factors and organizational profitability.

The current study used an observational study design, and despite robust statistical techniques, this limits its ability to establish a causal relationship between organizational health and productivity management and profitability. For example, the associations of higher per-employee costs for health services and medical services with increasing corporate profits may be explained by the fact that a company with increasing profits can provide better benefits for employees, including health insurance, tuition reimbursement, wellness stipends, and paid parental leave. Consequently, employees in a company with increasing profits may be less likely to have certain lifestyle-related health risk factors compared to employees in a company without increasing profits. The possibility of residual, unmeasured confounding also could not be excluded. The current study could not address whether the association of organizational health and productivity management with profitability is independent of factors that affect financial performance, including liquidity, ownership, age and size, leverage, solvency, and asset turnover [25,26].

Organizations’ management philosophy, policies, and systems were assessed using dichotomous yes/no questions in the survey. These survey questions were aggregated and coded for analyses as “conducted something= 1” for any “yes” answer or “conducted nothing= 0” for all “no” answers. Therefore, the companies in the “conducted something” group must be assumed to have used heterogeneous approaches. Further research is needed to identify specific health and productivity management strategies and systems that improve employees’ lifestyle-related health risk factors (i.e., systems that reduce smoking and improve sleep quality and exercise). These strategies and systems may not be identical across companies because of cultural differences across industries or due to company histories.

In this study of national health and productivity management programs, we demonstrated associations between employees’ lifestyle-related health risk factors and organizational profitability. By identifying and addressing health risks that impair worker performance, lifestyle medicine professionals may be able to provide a significant return on investment by creating a healthier and more productive workforce.

## DATA AVAILABILITY

We used survey responses from companies (i.e., aggregated data from multiple respondents from the same company to yield the company’s response), not individual participant data. Therefore, the survey data can be shared for purposes of reproducing the results or replicating the procedures by submitting a manuscript proposal to the Ministry of Economy, Trade and Industry (https://www.meti.go.jp/policy/mono_info_service/healthcare/kenko_keiei.html). The data will become available immediately following publication.

## Figures and Tables

**Table 1.** Characteristics at baseline

Characteristics	Company (n=1,593)
Age (yr)	40.3±3.4
Women	1,126,005 (25.8)
Length of service for employees (yr)	14.2±4.9
Type of industry	
Construction	69 (4.3)
Food	53 (3.3)
Chemistry	65 (4.1)
Electrical manufacturing	94 (5.9)
Transportation equipment	61 (3.8)
Shipping	55 (3.5)
Telecommunication	198 (12.4)
Wholesale	117 (7.3)
Retail	179 (11.2)
Financial services	139 (8.7)
Professional services	202 (12.7)
Other	361 (22.7)
Stock listing	793 (49.8)

Values are presented as mean±standard deviation or number (%).

**Table 2.** Confusion matrix for binary classification of a company with an increase in corporate profits and a company without an increase in corporate profits

Predicted values	Observed values		Total
	Positive (a company with an increase in corporate profits or sales)	Negative (a company without an increase in corporate profits or sales)	
Positive (a company with an increase in corporate profits or sales)	398	3	401
Negative (a company without an increase in corporate profits or sales)	1	1,191	1,192
Total	399	1,194	1,593

## SUPPLEMENTARY MATERIALS

Supplementary Material 1. Survey questions use d for the analysis

Supplementary Material 2 The distribution of an increase in corp orate profits from baseline to FY2020
