# Supplementary material for: The associations of the national health and productivity management program with corporate profits in Japan
Source: Epidemiol Health. 2022 Sep 23;44:e2022080. doi: 10.4178/epih.e2022080 (PMC10106540; doi:10.4178/epih.e2022080)
Supplement: Supplementary Material 1. — Survey questions use d for the analysis [file epih-44-e2022080-Supplementary-1.pdf]

**Supplementary Material 1. Survey questions used for the analysis**	
**Question**	**Response**
Q1. Is your company listed on a stock exchange?	Yes or No
Q2. Please select the type of industry in which your company operates.	Construction, food, chemistry, electrical manufacturing, transportation equipment, shipping, telecommunication, wholesale, retail, financial services, professional services, other
Q3. Please describe the number of full-time employees and part-time employees by sex and age.	Absolute numbers
Q4. Please describe the average age and length of service of full-time employees.	Absolute numbers
Q5. Please describe the number of full-time employees who left the organization during the fiscal year.※	Absolute number per 1,000 employees
Q6. Of the full-time employees in your company, how many persons are categorized as new employees and how many as mid-career hires?	Absolute number per 1,000 employees
Q7. What percentage of full-time employees in your company are assigned to the following roles?	Sales, research and development, design, project planner, marketing, production, manufacturing, distribution of goods, customer service, other
Q8. Please note corporate revenues and profits during the fiscal year※ in your company.	Absolute numbers
Q9. Do your company policies include a focus on health and productivity management?	Yes or No
Q10. Does your company communicate aims, organizational frameworks, and the content of your health and productivity management program, as well as achievements by the management?	Yes or No
Q11. Does your company share health and productivity management information with	Yes or No

investors?	
Q12. Does your company discuss health and productivity management goals and accomplishments during business management meetings and meetings of the board of directors?	Yes or No
Q13. In your company, who is assigned primary responsibility for health and productivity management?	President, board member, general manager, manager, section chief, regular employee, or none
Q14. Is responsibility for health and productivity management assigned to a specific department in your company?	Yes or No
Q15. Does your company share and discuss health and productivity management strategies with labor unions and employee representatives?	Yes or No
Q16. How many persons are involved in health and productivity management in your company?	Absolute numbers of staff members, occupational health physicians, public health nurses, nurses
Q17. Does your company discuss specific plans for health and productivity management with your health insurance association or medical insurers?	Yes or No
Q18. Does your company regularly use data to identify and improve issues related to health and productivity management?	Yes or No
Q19. What percentage of employees in your company have an annual medical check-up?	Percentage
Q20. What percentage of employees in your company have follow-up examinations after an annual health check-up?	Percentage
Q21. What percentage of employees in your company have a body mass index between 18.5 and 25 kg/m^2?	Percentage
Q22. What is the percentage of current smokers in your company?	Percentage
Q23. What is the percentage of employees in your	Percentage

company who have a habit of regular exercise?	
Q24. What is the percentage of employees in your company who sleep well?	Percentage
Q25. Does your company have specific strategies to improve the rate of participation in optional health examinations (e.g., a thorough medical checkup, cancer screening, hepatic virus screening)	Yes or No
Q26. Does your company have specific strategies to encourage employees to follow up with a doctor's office if they were recommended to seek treatment after receiving results from an annual medical check-up or optional health examination (e.g., a thorough medical checkup, cancer screening, hepatic virus screening)?	Yes or No
Q27. Does your company conduct a stress assessment for employees?	Yes or No
Q28. Does your company set and execute health and productivity management goals and tasks?	Yes or No
Q29. Does your company educate managers regarding how to pursue health and productivity management?	Yes or No
Q30. Does your company provide employees with education about health management topics, including improving mental health and preventing cancer?	Yes or No
Q31. Does your company have systems and strategies to optimize work schedules for employees?	Yes or No
Q32. Does your company provide information about coping strategies for employees who work longer hours beyond a standard schedule?	Yes or No
Q33. Does your company set criteria for interviewing employees who often work overtime?	Yes or No
Q34. Is your company aware of how many hours its full-time employees typically work?	Yes or No
Q35. Are there full-time employees who regularly	Yes or No

work overtime in your company?	
Q36. Does your company have specific strategies to promote communication between employees beyond just providing an instructional course, information, and banquets?	Yes or No
Q37. Does your company have specific strategies to support employees who are returning to work after experiencing mental health or other medical problems, and to balance work and medical treatment for employees?	Yes or No
Q38. Beyond health guidance in an annual medical check-up, does your company provide other health guidance to employees who are determined to be at increased risk for lifestyle-related diseases?	
Q39. Beyond health guidance in an annual medical check-up, does your company provide other health guidance to employees who are determined to be at increased risk for lifestyle-related diseases?	Yes or No
Q40. Based on the results from an annual medical check-up, do occupational physicians classify employees as being fit for regular work, having work-related restrictions, or requiring time off from work?	Yes or No
Q41. Does your company provide specific supports beyond just providing an instructional course and information to improve dietary habits for employees?	Yes or No
Q42. Does your company provide specific supports beyond just providing an instructional course and information to increase physical exercise for employees?	Yes or No
Q43. Does your company have other specific supports to improve employees' lifestyles?	Yes or No
Q44. Does your company have specific strategies for preventing infectious diseases, e.g., influenza?	Yes or No
Q45. Does your company restrict or ban smoking in	Yes or No

all domestic workplaces, including the head office?	
Q46. Does your company provide financial support for special medical examinations beyond annual medical check-ups?	Yes or No
Q47. Does your company provide any supports to employees' dependents to engage in health and productivity management?	Yes or No
Q48. With regard to health and productivity management, does your company provide facilities and supports for regular employees as well as temporary employees, workers from other companies, or workers with outsourced contracts?	Yes or No
Q49. When your company purchases products and services or arranges for work to be performed by your business partners, does your company consider the occupational safety and health of your business partner's employees?	Yes or No
Q50. Do industrial health physicians or nurses in your company engage in health and productivity management?	Yes or No
Q51. Does your company educate and train employees (including professionals) who are engaged in promoting and maintaining employee health and productivity?	Yes or No
Q52. Does your company assess the number of full-time employees who are absent from work, retired, or who died while in service or during absences from work?	Yes or No
Q53. Does your company evaluate the effects of health and productivity management on employees' health and productivity?	Yes or No
Q54. Does your company have specific metrics to evaluate the effects of health and productivity management on financial performance?	Yes or No
Q55. Does your company define the maintenance and promotion of employee health as a strategy for	Yes or No

business management?	
Q56. Does your company cooperate with other companies or participate in groups to implement health and productivity management?	Yes or No
Q57. Does your company utilize technologies (e.g., wearable devices, artificial intelligence, big data) to implement health and productivity management?	Yes or No
Q58. Has your company committed to invest ≥15,000 yen to maintain and promote employees' health in the next year?	Yes or No
Q59. What is your company's per-employee cost for medical and health services?	Absolute number
Q60. Please describe the amount of per-regular employee welfare expenses※※ in your company during the fiscal year※※※	Absolute number

※We selected survey questions that were similar in content between 2017 and 2018.

※※Welfare expenses include not only medical and health services, but also other services, including insurance for care, unemployment insurance, and industrial injury insurance.

※※※Fiscal year 2018 ran from April 2017 to March 2018, and fiscal year 2017 ran from April 2016 to March 2017.
