# Supplementary material for: The associations of the national health and productivity management program with corporate profits in Japan
Source: Epidemiol Health. 2022 Sep 23;44:e2022080. doi: 10.4178/epih.e2022080 (PMC10106540; doi:10.4178/epih.e2022080)
Supplement: Supplementary Material 2 — The distribution of an increase in corp orate profits from baseline to FY2020 [file epih-44-e2022080-Supplementary-2.pdf]

Figure S1

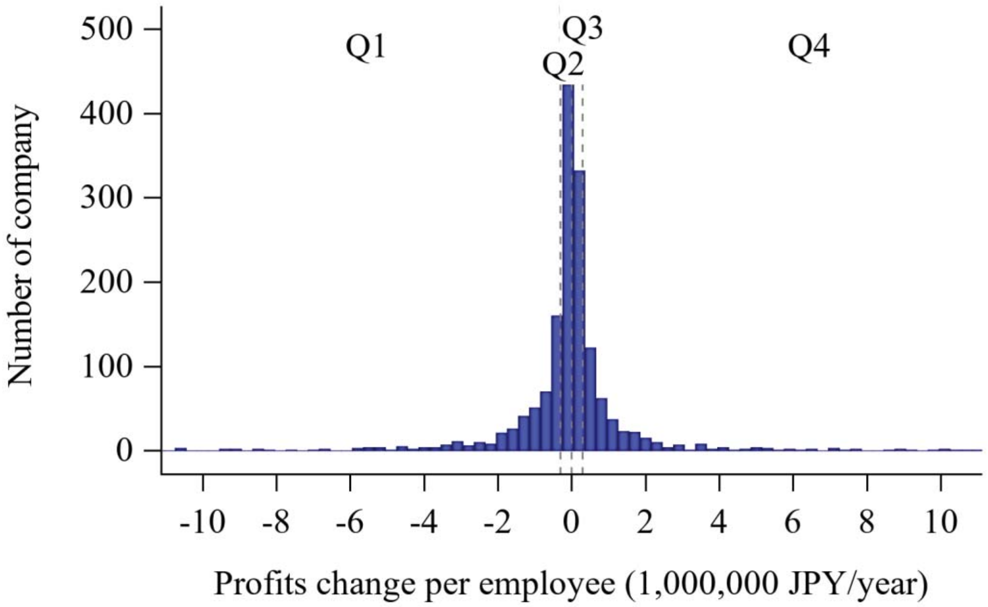

Supplementary Material 2. The distribution of an increase in corporate profits from baseline to FY2020

The distribution of an increase in corporate profits is shown. When we defined baseline data collected for FY2017 as the exposure, the rate of change in profits from FY2017 to FY2020 was calculated. When we defined baseline data collected for FY2018 as the exposure, the rate of change in profits from FY2018 to FY2020 was calculated. For each company, we calculated profit divided by the number of employees each year and evaluated the rate of change in profit per employee between baseline (2017 or 2018) and 2020. We categorized all companies into quartiles based on rate of change in profits after baseline and defined the highest-quartile group as comprising companies that had an increase in corporate profits. FY= fiscal year
